# Metabolome Alterations Associated with Three-Month Sitting-Time Reduction Among Sedentary Postmenopausal Latinas with Cardiometabolic Disease Risk

**DOI:** 10.3390/metabo15020075

**Published:** 2025-01-26

**Authors:** Jeffrey S. Patterson, Paniz Jasbi, Yan Jin, Haiwei Gu, Matthew A. Allison, Chase Reuter, Brinda K. Rana, Loki Natarajan, Dorothy D. Sears

**Affiliations:** 1 College of Health Solutions, Arizona State University, Phoenix, AZ 85004, USA; jspatte5@asu.edu (J.S.P.);; 2 School of Molecular Science, Arizona State University, Phoenix, AZ 85004, USA; 3 Department of Family Medicine, University of California San Diego, La Jolla, CA 92037, USA; 4 Department of Psychiatry, University of California San Diego, La Jolla, CA 92037, USA; 5 Herbert Wertheim School of Public Health and Human Longevity Science, University of California San Diego, La Jolla, CA 92037, USA; 6 Moores Cancer Center, University of California San Diego, La Jolla, CA 92037, USA

**Keywords:** sedentary behavior, aging, endothelial function, blood flow, glycemic control, mitochondria, Hispanic, branched-chain amino acids, acylcarnitines, TMAO, control group effects

## Abstract

**Background:** Incidence of cardiometabolic disease among U.S. Hispanics/Latinos is higher than in non-Hispanic Whites. Prolonged sitting duration is prevalent in older adults, and compounded with menopause, greatly increases cardiometabolic risk in postmenopausal women. Metabolomic analyses of interventions to reduce sitting are lacking and mechanistic understanding of health-promoting behavior change in postmenopausal Latinas is needed. **Methods:** To address this knowledge gap, an exploratory analysis investigated the plasma metabolome impact of a 12-week increased standing intervention among sedentary postmenopausal Latinas with overweight or obesity. From a parent-randomized controlled trial, a subset of Best Responders (*n* = 43) was selected using parameters of highest mean change in sitting bout duration and total sitting time; baseline variable-Matched Controls (*n* = 43) were selected using random forest modeling. Targeted LC-MS/MS analysis of archived baseline and 12-week plasma samples was conducted. Metabolite change was determined using a covariate-controlled general linear model and multivariate testing was performed. A false discovery rate correction was applied to all analyses. **Results:** Best Responders significantly changed time sitting (−110.0 ± 11.0 min; −21%), standing (104.6 ± 10.1 min; 40%), and sitting in bouts >30 min (−102.3 ± 13.9 min; −35%) compared to Matched Controls (7.1 ± 9.8 min, −7.8 ± 9.0 min, and −4.6 ± 12.7 min, respectively; all *p* < 0.001). Twelve-week metabolite change was significantly different between the two groups for 24 metabolites (FDR < 0.05). These were primarily related to amino acid metabolism, improved blood flow, and ATP production. Enzyme enrichment analysis predicted significant changes regulating glutamate, histidine, phenylalanine, and mitochondrial short-chain fatty acid catabolism. Pathway analysis showed significant intervention effects on glutamate metabolism and phenylalanine, tyrosine, and tryptophan biosynthesis, potentially indicating reduced cardiometabolic disease risk. **Conclusions:** Replacing nearly two hours of daily sitting time with standing and reduced prolonged sitting bouts significantly improved metabolomic profiles associated with cardiometabolic risk among postmenopausal Latinas.

## 1. Introduction

Nearly half of American Hispanic/Latinas are overweight or obese, which is significantly higher than non-Hispanic Whites and increases risk of adverse health outcomes [1,2]. In fact, the US Hispanic/Latino population is currently at a 1.5-fold increase in mortality due to diabetic complications [3]. The incidence of cardiometabolic disease is further exacerbated in Hispanic/Latina postmenopausal women due to age- and menopause-associated metabolic alterations, and is evidenced by twice the prevalence of having one or two chronic diseases [4,5,6]. Menopause results in physiological alterations like decreased estrogen and increased androgen, which diminish energy metabolism and increase abdominal obesity [7]. Furthermore, visceral adipose tissue breakdown increases circulating free fatty acids and raises the risk of developing insulin resistance [8]. These metabolic effects place postmenopausal Hispanic/Latina women at an even greater risk of inflammation, dyslipidemia, glucose dysregulation, and type 2 diabetes mellitus (T2D) [9].

Sitting for long periods of the day has become commonplace in the aging population due to a variety of physical, environmental, and social factors [10]. Prolonged periods of daily sitting time are associated with an increased risk of obesity, T2D, cardiovascular disease (CVD), and mortality [11]. Epidemiologic studies have also shown correlations of greater sitting time and poor cardiometabolic health outcomes, such as increased adiposity and waist circumference, insulin resistance, and diminished physical function in older adults [12,13,14]. Indeed, a recent investigation of the Hispanic Community Health Study/Study of Latinos (HCHS/SOL) cohort found that prolonged, uninterrupted sedentary behavior had a significant dose-dependent relationship with the Homeostatic Model Assessment for Insulin Resistance (HOMA-IR) and 2 h postprandial glucose concentrations [15]. Moreover, a recent analysis of objectively measured mean sitting bout duration and fasting glucose, insulin, and triglycerides in over 500 postmenopausal women suggests that the association may be more deleterious in those of Hispanic/Latina descent than in those who are non-Hispanic [14].

Prolonged sitting during the day results in significantly impaired vascular function in the lower extremities [16,17]. As a result, extensive sitting time is associated with and physiologically involved in cardiometabolic disease development. Rising to stand from a seated position immediately raises circulation and enhances nutrient, insulin, and oxygen delivery to the lower extremities [17,18,19]. Acute standing breaks during prolonged sitting bouts improve vascular function and glycemic control [17,20,21]. Replacing sitting with standing is a practical and feasible long-term behavior change that may improve health outcomes in individuals who sit for prolonged periods.

Metabolomics enables quantification of low molecular weight substrates and products of metabolism to assess the current physiological function of metabolic pathways [22,23,24,25]. Mass spectrometry (MS)-based metabolic profiling has been widely employed for a variety of purposes in investigative pathway analyses, disease diagnosis, and drug development [26]. Examining the plasma metabolome can uncover altered physiological pathways present in cardiometabolic disease risk [27,28,29,30]. Risk of incident insulin resistance, T2D, and CVD has been identified years before onset through the identification of altered metabolic intermediary profiles of mitochondrial pathways [31,32,33,34,35,36]. Previous studies have shown success in interventions to increase standing, but little is known about its impact on metabolite profiles [17,37]. Only a few metabolomic studies have investigated the metabolic signatures of prolonged sitting and replacing sitting with standing [38,39,40,41]. We aim to advance the current mechanistic knowledge of the metabolic impacts elicited by prolonged sitting behavior interventions. As such, we explored the plasma metabolome alterations induced by a 12-week intervention that replaced sitting with standing delivered in a randomized controlled trial among sedentary postmenopausal Latinas with elevated cardiometabolic risk.

## 2. Methods

### 2.1. Participants

The *Arriba por la Vida Estudio* (AVE) parent study protocol, from which the archival plasma sample data was obtained, has been previously published [37,42]. A two-arm randomized controlled trial (RCT) was approved by the University of California, San Diego (UCSD) Institutional Review Board. Participants provided written informed consent during the screening visit. The parent clinical trial was the Clinical Science Project component of an AHA-funded Strategically Focused Research Network (SFRN) Go Red for Women Center. This exploratory metabolomics analysis of biospecimens collected in the Clinical Science Project was a primary aim of the Basic Science Project component of the center.

Sedentary postmenopausal Latinas with overweight or obesity (*n* = 254; 55 + y; BMI ≥ 25 kg/m^2^) were randomized to an increased standing intervention or a heart-healthy lifestyle education control. For the current metabolomics analysis, our interest was the identification of metabolome alterations associated with replacing sitting with standing. We speculated that the intervention group participants that achieved the greatest reduction in sitting behavior would have identifiable metabolome alterations associated with that behavior change. To this end, a subsample of 86 participants was selected from the total parent RCT sample. We applied a linear mixed model analysis (with random intercept and slope) to sitting behavior data collected from the intervention group participants to identify those in the top tertile of sitting behavior change. We refer to these top tertile participants as “Best Responders” (*n* = 43). We then developed a random forest model to predict Best Responders’ status (Y or N) using baseline covariates. Cross-validation was used for hyper parameter tuning. The final random forest model was then applied to the control group to obtain predicted probabilities of being in the Best Responder class. The 43 participants in the control group with the highest predicted probabilities were then selected as the “Matched Controls”. This approach for selecting Matched Controls allowed us to maintain the randomized design for the metabolomics analysis. Daily sitting, standing, physical activity, sleep behaviors and beliefs, body composition, education, income, short physical performance battery score, and perceived stress, anxiety, and well-being were used in the model. The only significant group differences were in the variables concerning the patient health questionnaire (PHQ2), brief resilience scale (BRS6), well-being (CESD10), and general anxiety disorder (GAD7).

### 2.2. Parent Study Design

Study design details have been previously published [37,42]. Sedentary postmenopausal Latinas were randomized to an increased standing intervention or a heart-healthy lifestyle education control. The intervention group was asked to increase standing time in additional increments of thirty minutes in five-minute bouts per day for the first four weeks with the goal of obtaining two hours of increased daily standing in week four. The participants were then asked to maintain the two-hour daily standing goal for the remaining eight weeks of the study. The control group was provided heart-healthy lifestyle counseling that included stress, sleep, bladder control, home safety, hydration, depression, aging, and medication management counseling (Table S1). All standing time was assessed objectively by a thigh-worn inclinometer and fasting plasma measurements were collected at baseline and at 12 weeks.

*Standing Intervention*—The standing intervention group (Intervention) was provided three in-person health counseling sessions with a certified health coach located in a community-based research center, a single home visit, and as many as five telephone calls with the coach.

*Heart-Healthy Lifestyle Comparison*—The heart-healthy lifestyle comparison group (Control) was provided an initial in-person health counseling session from a certified health coach located in a community-based research center and was followed by 12 weekly thirty-minute counseling sessions by telephone focused on healthy aging lifestyle changes.

### 2.3. Metabolomics

Archival plasma samples from the parent study were collected at baseline and at 12-week follow-up. A targeted approach using high-performance liquid chromatography mass spectrometry (HPLC-ESI MS/MS) of nearly 300 metabolites, including a mitochondrial function panel of predictive metabolites of incident cardiac events and T2D (amino acids, acylcarnitines, and organic acids), was conducted at the Arizona Metabolomics Laboratory.

#### 2.3.1. Reagents

LC-MS grade Acetonitrile (ACN), methanol (MeOH), ammonium acetate (NH_4_OAc), and acetic acid (AcOH) were obtained from Fisher Scientific (Pittsburgh, PA). Ammonium hydroxide (NH_4_OH) was purchased from Sigma-Aldrich (Saint Louis, MO, USA). DI water was obtained in-house by a Water Purification System from EMD Millipore (Billerica, MA, USA). GE Healthcare Life Sciences (Logan, UT, USA) provided phosphate-buffered saline (PBS). Standard compounds analogous to the quantified metabolites were bought from Sigma-Aldrich (Saint Louis, MO, USA) and Fisher Scientific (Pittsburgh, PA, USA).

#### 2.3.2. Sample Preparation

The sample preparation protocol was modeled from previous studies [43]. Frozen plasma samples were thawed overnight under 4 °C, and 50 μL of each sample was transferred to a 2 mL Eppendorf vial. Protein precipitation and metabolite extraction was executed by adding 300 μL of methanol containing ^13^C_6_-Glucose and ^13^C_5_-Glutamic acid. The solution was then vortexed for 2 min and stored at −20 °C for 30 min. The solution was sonicated in an ice bath for 10 min with subsequent centrifugation at 14,000 RPM for 20 min at 4 °C. The supernatant (150 μL) was transferred into a new Eppendorf vial, and dried using a Vacufuge Plus evaporator. The dried samples were reconstituted in 500 μL of 5 mM ammonium acetate in 40% H_2_O/60% ACN + 0.2% 300 acetic acid with 5.13 μM L-tyrosine-^13^C_2_ and 22.5 μM sodium-L-lactate-^13^C_2_. The stable isotope-labeled internal standards were then placed in each sample to monitor system performance. A pooled sample, a plasma mixture from all participants, was prepared using the same procedure as previously described. This pooled sample served as a quality control (QC) purpose and was measured once every 10 study samples.

#### 2.3.3. Liquid Chromatography and Mass Spectrometry Conditions

The targeted LC-MS/MS method used here was modeled and has been published in a growing number of studies [29,43,44]. LC-MS/MS experiments were briefly performed on a Waters Acquity I-Class UPLC TQS-micro-MS system. Each sample was injected twice with 2 μL for positive and 5 μL for negative ionization mode. Chromatography separation was obtained with a Waters Xbridge BEH Amide column (2.5 μm, 2.1 × 150 mm) at 40 °C with a flow rate of 0.3 mL/min. For positive ionization, the mobile phase was comprised of Solvents A (5 mM ammonium acetate in H_2_O with 0.1% acetic acid) and B (ACN with 0.1% acetic acid). For negative ionization, Solvent A was 10 mM ammonium bicarbonate in H_2_O, and Solvent B was ACN. The LC gradient conditions were held the same for both ionization modes. Following the initial 1.5 min isocratic elution of 10% Solvent A, the percentage of Solvent A was increased linearly to 65% at t = 9 min. The percentage of A was then held the same (65%) for 5 min (t = 14 min), after which the percentage of A was decreased to 10% at t = 15 min to prepare for the next injection. The total experiment time for each injection was 30 min.

### 2.4. Statistical Analyses

Linear mixed models with subject-level random intercepts and slopes were used to examine sitting behavior outcome change between the Best Responders and Matched Controls. Metabolite ratios were calculated as follow-up/baseline using Microsoft Excel (Redmond, WA, USA). A general linear regression analysis utilizing covariates PHQ2, BRSS6, CESD10, and GAD7 to control for between-group differences was conducted for metabolite change using IBM SPSS Statistics 27 (Armonk, NY, USA). Partial least squares-discriminant analysis (PLS-DA), variable importance projection (VIP) scores, pathway and enzyme enrichment analyses, and a heat map were log-transformed and analyzed between conditions using the MetaboAnalyst 6.0 software [45]. A threshold α-level of 0.05 was used to define statistical significance between groups and a false discovery rate (FDR) was applied to all analyses.

## 3. Results

### 3.1. Participant Characteristics

In this ancillary exploratory analysis using plasma collected during the parent clinical trial [37], we aimed to identify metabolome alterations associated with sitting behavior change. Thus, parent study intervention group participants exhibiting the greatest change in sitting behavior were selected for the current analysis and termed “Best Responders” (*n* = 43). Best Responders were selected by the criteria of being in the top tertile of change in mean sitting bout duration and total sitting time. Baseline variable-Matched Controls (*n* = 43) were selected from the parent study control group participants using a random forest model. Baseline characteristics and variables used in the model are shown in Table 1. This model was used to identify parent study control group participants predicted to result in similar mean sitting bout duration change and total sitting time, if they had been randomized to the intervention.

Sitting behavior outcomes were significantly different between Best Responders and Matched Controls (Table 2). After 12 weeks of intervention, the Best Responders reduced daily sitting time by 21%, increased total standing time by 40%, and increased daily stepping time by 7%. In addition, this group reduced daily time spent in sitting bouts of thirty minutes or greater by 35% and mean sitting bout duration by 20% as well as increased moderate to vigorous physical activity by 29%.

### 3.2. Analysis of 12-Week Metabolite Change

Plasma metabolite measurements generated from baseline and 12-week follow-up (intervention end point) time points were analyzed for change. Metabolite changes were compared between groups. After FDR correction, general linear models identified 24 metabolites for which change was significantly different between the Best Responders of the standing intervention group and the Matched Controls of the heart-healthy lifestyle comparison control group (Table 3). Metabolites identified included those associated with amino acid metabolism (valine, norvaline, histidine, leucine, isoleucine, tyrosine, adenosyl-L-homocysteine, kynurenine, 5-hydroxyindoleacetic acid, and 2-aminoadipic acid). In addition, molecules linked to sugar metabolism or energy production (pyruvate, glucose, fructose, mannose, and oxoglutaric acid) and lipid metabolism (propionyl-L-carnitine, 2-methylbutyrl-L-carnitine, isovaleryl-L-carnitine, isobutyric acid, and 2-methylglutaric acid) also demonstrated significant group differences. Additional compounds of note were trimethylamine N-oxide (TMAO), epinephrine, acetohydroxamic acid, and N,N-dicyclohexylurea. A total list of non-FDR corrected significant metabolites between groups is shown in Table S2.

**Figure 1:**
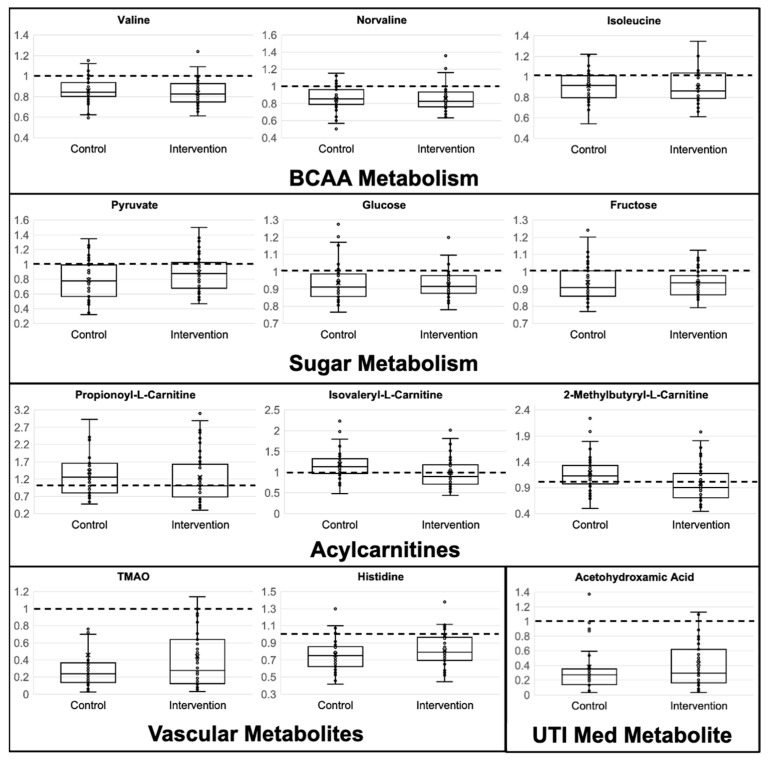
Metabolites of special interest identified in general linear model as significantly different in Best Responders standing intervention and the Matched heart-healthy lifestyle comparison Control Fold Change Differences. Y-axis shows fold change, calculated as 12-week follow-up/baseline measurements. X—mean, line—median, circles—individual samples. Fold change of 1.0 means no change from baseline to 12-week follow-up. Horizontal dashed line drawn at fold change of 1.0.

Fold change analysis (12-week follow-up divided by baseline) demonstrated significantly reduced metabolites associated with branched-chain amino acid (BCAA) metabolism at follow-up (FC < 1.0) in the Best Responders of the standing intervention group compared to the Matched Controls (Figure 1). Metabolites aiding in long-chain fatty acid β-oxidation (acylcarnitines) were reduced or unchanged after the standing intervention, while the Matched Controls were increased at follow-up (FC > 1.0). Metabolites related to sugar metabolism, vascular function, and urinary tract infections (UTI) were reduced at follow-up in both groups, potentially indicating similarly positive effects from increased standing and the heart-healthy education.

**Figure 2:**
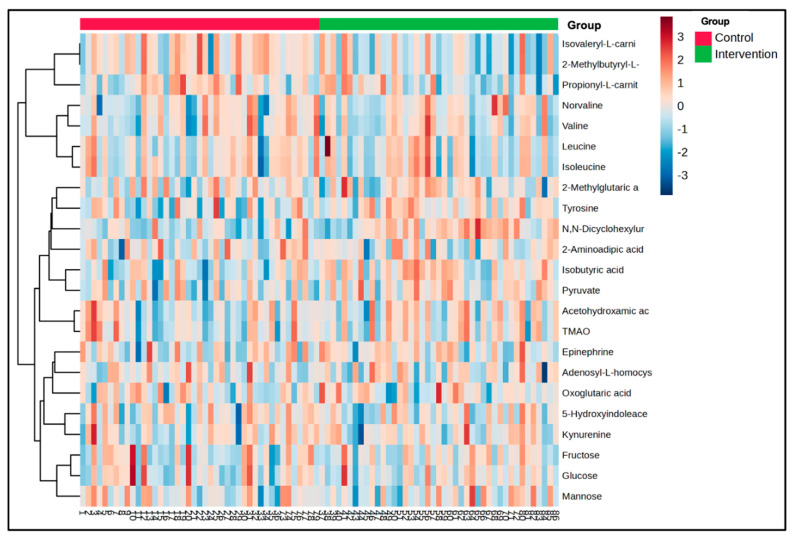
Heat map of metabolite fold change for which 12-week change was significantly different between Best Responders standing intervention and the Matched heart-healthy lifestyle comparison Control (*p* < 0.05). Display demonstrates normalized relative increases and decreases in abundance between and within conditions among postmenopausal women.

A heat map showing normalized mean relative fold change in abundance across the groups was generated to illustrate the directionality of within group change (12-week follow-up/baseline) for each of the 24 significant metabolites (Figure 2). Metabolite change scores mapped to central carbon metabolism, with significant alterations to metabolites associated with energy production (glycolysis, sugar metabolism, and carnitine derivatives), increased blood flow (norvaline), and tryptophan metabolism (5-hydroxyindoleacetic acid and kynurenine). A heat map depicting both time and group differences of all significant metabolites is shown in Figure S1.

**Figure 3:**
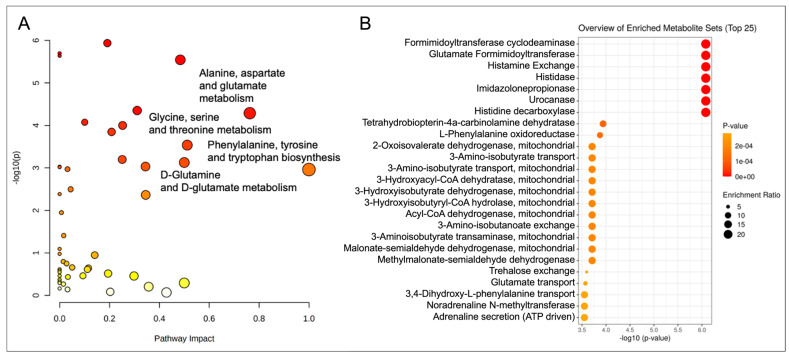
Pathway (**A**) and enzyme enrichment (**B**) analyses of the Best Responders standing intervention and the Matched heart-healthy lifestyle comparison Control fold change differences. Data is plotted as −log_10_(*p*) versus pathway impact. The darker red color depicts greater significance and the larger circle size represents increased enrichment.

Pathway analysis of the Best Responders standing intervention fold change and the Matched Controls heart-healthy lifestyle comparison control fold change showed significant group effects on amino acid metabolism, including phenylalanine, tyrosine, and tryptophan biosynthesis, and alanine, aspartate, and glutamate metabolism (*p* < 0.05, Impact > 0.05) (Figure 3A). In addition, glycine, serine, and threonine metabolism, and D-glutamine and D-glutamate metabolism were significant between groups (*p* < 0.05, Impact > 0.05). Enzyme activity enrichment predicted significant changes in glutamate and histidine metabolism as well as mitochondrial short-chain fatty acid breakdown (*p* < 0.05, Enrich > 10) (Figure 3B).

**Figure 4:**
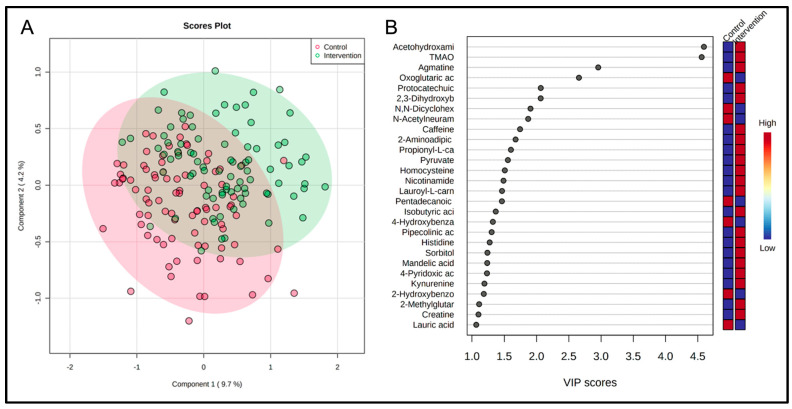
PLS-DA score plot (**A**) and variable importance projection scores (**B**) comparing Best Responders standing intervention and the Matched Controls heart-healthy lifestyle comparison control fold change differences. Each dot in the PLS-DA score plot (**A**) represents a fold change (12-week follow-up/baseline) of Best Responder intervention and Matched Control samples. The directionality and influence of metabolites are depicted as a VIP score (**B**).

Partial least squares-discriminant analysis demonstrated significance between group differences in metabolite clustering for the Best Responders standing intervention and Matched Controls fold change scores (Figure 4A). Distinct group clustering was depicted by variable importance projection (VIP) scores > 1.0 (Figure 4B). The score plot was primarily driven by acetohydroxamic acid, TMAO, metabolites associated with energy production (carnitine derivatives, oxoglutaric acid, pyruvate, and nicotinamide), N,N-dicyclohexylurea, amino acids (homocysteine and histidine), and kynurenine. Cross-validation testing was performed to ensure proper model fit and is shown in (Figure S2).

## 4. Discussion

Although the accumulated evidence demonstrates the benefit of interrupting prolonged sitting with low-intensity interruptions like standing [17,37], little is known about how such interventions and prolonged sitting impact the metabolome [38,39,40,41]. Our aim was to identify metabolites and metabolic pathways that are responsive to increased standing and indicative of improved cardiometabolic health. The parent study 12-week intervention was successful in significantly reducing sitting time and sitting bout duration while significantly increasing standing, stepping, and moderate to vigorous physical activity among postmenopausal Latinas with cardiometabolic risk [37]. This exploratory metabolomics analysis investigated the plasma metabolome of intervention group participants who changed their sitting behavior the most, i.e., Best Responders, and baseline variable-Matched Controls using a targeted approach. Our analysis included a sample of 86 individuals, which is 5.7 times larger than any other sitting intervention metabolomics study to date. Alterations in 24 metabolites associated with branched-chain amino acid catabolism, tryptophan metabolism, energy production, and related pathways, suggest intervention-mediated cardiometabolic risk reduction.

Approximately fifty percent of U.S. Hispanic/Latinas have overweight or obesity, which is significantly higher than non-Hispanic Whites and increases health complication risk [1,2]. Cardiometabolic disease incidence is further exacerbated in Hispanic/Latina postmenopausal women due to age-related metabolic alterations [4,5,6]. These physiological effects place Hispanic/Latina postmenopausal women at a greater risk of conditions like inflammation, dyslipidemia, glucose dysregulation, and T2D [9]. In addition, longer bouts of daily sitting have become prevalent in the aging population due to a variety of factors [10]. Extended periods of daily sitting time are associated with an increased risk of obesity, T2D, CVD, and mortality [11]. Recent studies have shown correlations of greater sitting time and worse cardiometabolic outcomes like increased adiposity and waist circumference, insulin resistance, and diminished physical function [12,13,14].

Extensive sitting during the day results in significant vascular dysfunction in the lower extremities [16,17]. As a consequence of ninety-degree arterial bending at the hips and knees, extended sitting bouts alter hydrostatic pressure and hemodynamics, impacting transport and balance of blood gases, metabolites, and signaling molecules such as insulin [46]. Consequently, prolonged sitting time is associated with and mechanistically involved in the development of cardiometabolic disease. The practical method of replacing sitting with standing is a long-term behavior change that may improve health outcomes in those who sit for prolonged periods. Standing from a seated position immediately increases circulation in the lower extremities and raises shear stress on vascular endothelial cells, which improves intracellular signaling and reduces oxidative stress [18,19]. The increased blood flow from standing also enhances oxygen, nutrient, and insulin delivery to the lower limbs [17]. Acute standing breaks in prolonged sitting enhance glycemic control and vascular function [17,20,21].

The parent study 12-week intervention resulted in similarly altered plasma amino acids in the Best Responders, as did recent studies of prolonged vs. interrupted sitting in older adults [38,40,41]. Significant reductions in branched-chain amino acids (BCAAs) in plasma and muscle tissue metabolomes associated with MVPA were observed. The congruence between studies suggests that replacing sitting with standing in postmenopausal women at risk of cardiometabolic disease may similarly increase energy expenditure and provide beneficial effects. Elevated levels of circulating BCAAs have been widely reported to be associated with cardiometabolic dysfunction-related conditions like insulin resistance, T2D, atherosclerosis, CVD, and cancer [47,48,49,50]. Recent observational evidence suggests that altered BCAA catabolism is associated with dysregulation of crucial metabolic processes related to glycemic control [47,48,49,50]. Similar to the current findings, levels of norvaline, a BCAA derivative, were significantly impacted in our recently published study investigating the acute metabolome impacts of standing breaks in prolonged sitting time among postmenopausal women enrolled in a crossover trial [41]. Norvaline and its derivatives are potent nitric oxide inducers shown to have blood sugar- and blood pressure-reducing properties [51,52]. The physiological benefits of replacing sitting with standing demonstrate trends that coincide with reduced risk of cardiometabolic disease.

Hallmarks of energy expenditure and substrates of energy producing pathways were also significantly affected at follow-up. Compounds related to glycolysis and the tricarboxylic acid (TCA) cycle, pyruvate, glucose, and oxoglutaric acid, respectively, were reduced after the intervention, but significantly elevated compared to the control. A potential mechanism is that 12 weeks of increased standing improved fasting metabolite concentrations, but due to increased metabolic activity and demand, these substrates are higher than in the control group [53,54]. In addition, acylcarnitine concentrations were largely reduced or unchanged from the baseline after replacing sitting with standing. Acylcarnitines are vital proteins tasked with shuttling long-chain fatty acids across the mitochondrial membrane for β-oxidation and energy production [55]. Low plasma acylcarnitine levels are considered to be evidence of good mitochondrial function, while high circulating levels have been widely associated with insulin resistance and T2D risk [56,57,58]. The comparison control displayed increases from baseline in all acylcarnitine derivatives, potentially indicating the negative cardiometabolic effects of prolonged sitting.

Increased standing as a modality to reduce long sitting bouts may also have far reaching implications related to and independent of metabolism. A pathway analysis of both groups revealed significantly altered tryptophan metabolism. Tryptophan has two central pathways and is a precursor to kynurenine and serotonin [59,60]. The intervention resulted in significantly lower 5-hydroxyindoleacetic acid, a waste product of serotonin, and kynurenine levels, potentially indicating a more active serotonin pathway with less serotonin turnover. A well-known hormone, epinephrine, was also elevated compared to the control, and may highlight the effects of increased standing. Also referred to as adrenaline, epinephrine is released in response to stress and physical activity [61,62]. The pathway analysis also uncovered other alterations to amino acid metabolism like glutamate metabolism, which was also represented in the enrichment analysis of estimated enzyme activity. Glutamate was considerably lower after the intervention and compared to the control. High levels of plasma glutamate have been associated with metabolic dysfunction and insulin resistance due to a dysregulated glutamate/receptor axis and insulin secretion in pancreatic β-cells [63,64]. Increased physical activity has shown to reduce circulating glutamate and potentially improve insulin sensitivity [64]. However, glutamate can also be formed by the breakdown of histidine via histidase and urocanase [65], which were significantly enriched along with histamine exchange and histidine decarboxylase. In response to exercise, histamine is released in the lower extremities to promote blood flow, and has been associated with rate of femoral artery blood flow [66,67] In addition, several mitochondrial enzymes involved in short-chain fatty acid breakdown were identified. A partial least squares-discriminant analysis also confirmed several important metabolites in differentiating the two groups, such as TMAO, energy-producing compounds like the carnitine derivatives, oxoglutaric acid, pyruvate, and nicotinamide, as well as histidine and kynurenine. The cardiometabolic effects of replacing sitting with standing appear to be distinct for all analyses.

A heart-healthy lifestyle education provided to control group participants included weekly coaching in improving hydration, bladder control, stress, depression, and sleep quality. The education program was offered as a “touch control” and an incentive to reduce attrition. Typically, ‘one time per topic’ education coaching is not expected to impact behavior long-term. In contrast, our metabolomics results from the Matched Controls indicate the education impacted their behavior. For example, Matched Controls’ significant reductions in plasma levels of glucose, fructose, and a medication used to treat urinary tract infections (acetohydroxamic acid) may have been related to coaching about hydration, bladder control, and medication management. A novel method to match a subset of controls to the Best Responders from the parent study was used to identify control subjects that would likely have been best responders if they had been randomized to the intervention group. It would be fair to postulate that the Matched Control participants exhibited the same best responding behavior with the heart-healthy lifestyle coaching and modified daily habits, observable at the metabolome level at follow-up. Many of the metabolites significantly changed in the standing intervention Best Responders were similarly affected in the Matched Controls group. Unexpected shifts in control participant behavior can compromise between-group analyses and mask intervention effects. A nontrivial finding of our analysis is that metabolomics can identify unexpected control participant behavior change.

Despite many careful considerations and safeguards, the present study has several limitations. Although this study was the largest metabolomics analysis of sitting interventions to date, it is an ancillary study of archival plasma and was not powered to identify significant alterations in metabolite concentrations. This study revealed behavior change that occurred in the Matched Control group as a result of the heart-healthy lifestyle education. In future studies, material provided to control participants should be carefully considered to be neutral in behavior impact at follow-up or be presented to all participants. Ideally, the entire study population would have been analyzed rather than a subset of Best Responders and Matched Controls to provide a more comprehensive outcome and more statistical power. Clinical cardiometabolic parameter changes (blood pressure, body mass index, etc.) are not reported here and will be reported separately. Baseline BMI was not different between groups (Table 1). Lastly, this study examined the effects of increased standing in a postmenopausal Latina population and the results may not be generalizable to other race, ethnicity, and age groups. Future studies may include more diverse cohorts to verify the broader applicability of the findings. Further research on replacing sitting with standing is still needed to fully understand effects on the metabolome and cardiometabolic risk.

## 5. Conclusions

Applying a targeted metabolomics approach to plasmas collected in the course of an RCT testing a 12-week standing intervention to replace sitting revealed significant metabolite changes primarily related to amino acid metabolism, improved blood flow, and energy production. Enzyme enrichment analysis predicted significant enzymatic activity changes regulating glutamate, histidine, and mitochondrial short-chain fatty acid catabolism. Pathway analysis showed significant intervention effects on glutamate metabolism and tryptophan metabolism. Replacing nearly two hours of daily sitting time with standing alters metabolomic profiles associated with reduced cardiometabolic risk among postmenopausal Latinas. These findings may help guide future behavior change-associated biomarker identification as well as public health initiatives aimed to reduce cardiometabolic disease risk in older populations by interrupting prolonged sitting.

## Figures and Tables

**Table 1:** Ancillary study participant baseline characteristics and variables used for Matched Controls selection.

Variable	Matched Controls (*n* = 43)	Best Responders (*n* = 43)	*p*-Value	Variable	Matched Controls (*n* = 43)	Best Responders (*n* = 43)	*p*-Value
Age (y)	64.6 (7.5)	63.8 (7.2)	0.61	VOS (avg)	3.8 (0.7)	3.8 (0.6)	0.81
MVPA (min/d)	48.7 (17.3)	45.49 (13.1)	0.34	IWO (avg)	3.7 (0.4)	3.6 (0.4)	0.29
Stepping (min/d)	89.1 (23.8)	84.2 (22.0)	0.32	CASB (avg)	4.3 (0.5)	4.3 (0.6)	0.85
Sitting (min/d)	527.7 (72.1)	529.2 (77.5)	0.93	VASB (avg)	2.9 (0.4)	2.9 (0.5)	0.87
Sit bout duration (min)	13.0 (3.1)	13.2 (3.3)	0.86	SLEEP (min)	434.7 (83.9)	406.4 (110.3)	0.19
Waist Circ. (cm)	100.1 (11.7)	99.5 (10.8)	0.80	Sleep habits (avg)	1.7 (0.5)	1.9 (0.5)	0.10
BMI (kg/m^2^)	31.72 (4.86)	32.20 (4.13)	0.624	PSS4 (avg)	7.14 (2.47)	7.95 (2.59)	0.14
Education (y)	10.12 (4.35)	9.91 (4.51)	0.827	GPSS4 (avg)	6.72 (2.20)	7.30 (2.42)	0.246
Income ($)			0.874	**PHQ2 (avg)**	**2.40 (0.73)**	**3.05 (1.19)**	**0.003**
≤15K	18 (41.9)	17 (39.5)		**BRS6 (avg)**	**12.79 (3.05)**	**15.12 (3.59)**	**0.002**
>15K & ≤25K	9 (20.9)	12 (27.9)		**CESD10 (avg)**	**16.79 (2.04)**	**18.49 (4.23)**	**0.02**
>25K & ≤50K	13 (30.2)	12 (27.9)		**GAD7 (avg)**	**8.53 (2.20)**	**10.49 (3.34)**	**0.002**
>50K	3 (7.0)	2 (4.7)		LEC (avg)	5.35 (1.54)	5.12 (1.68)	0.505
				SPPB score	9.09 (1.84)	9.12 (1.35)	0.947

Note. Data are means ± SD. *p*-values generated from group comparisons were considered significant at <0.05. MVPA—moderate to vigorous exercise, BMI—body mass index, VOS—views on standing, IWO—interaction with others, CASB—confidence about standing breaks, VASB—views about sedentary behavior, SLEEP—sleeping minutes, PSS4—perceived stress scale, GPSS4—global perceived stress scale, PHQ2—patient health questionnaire, BRS6—brief resilience scale, CESD10—well-being, GAD7—general anxiety disorder, LEC—life events checklist, and SPPB—short physical performance battery.

**Table 2:** Sitting behavior outcomes in standing intervention Best Responders and Matched Controls.

Sitting Behavior Outcomes	Matched Controls Baseline	Matched Controls Change	Best Responders Baseline	Best Responders Change	*p*-Value	Percent Change
Sitting time (min/d)	514.9 (10.4)	7.1 (9.8)	526.0 (10.4)	−110.4 (11.0)	<0.0001	−21%
Standing time (min/d)	272.0 (9.7)	−7.8 (9.0)	261.0 (9.7)	104.6 (10.1)	<0.0001	+40%
Stepping time (min/d)	90.5 (1.8)	0.9 (1.3)	89.8 (1.8)	6.5 (1.5)	0.0045	+7%
Sitting in 30+ minute bouts (min/d)	291.4 (11.6)	−4.6 (12.7)	290.9 (11.5)	−102.3 (13.9)	<0.0001	−35%
Sitting bout duration (min/d)	12.8 (0.5)	0.3 (0.5)	12.9 (0.5)	−2.6 (0.5)	<0.0001	−20%
MVPA (min/d)	47.8 (2.7)	2.5 (2.2)	45.7 (2.6)	13.3 (2.2)	0.0008	+29%

Note. Baseline and change values mean ± SD. *p*-values generated from group change comparisons and were considered significant at <0.05. MVPA—moderate to vigorous physical activity. Percent change—change as a percent of baseline for Best Responders.

**Table 3:** Significant metabolite change between Best Responders standing intervention and the Matched Controls heart-healthy lifestyle comparison after 12 weeks.

Metabolite		*p*-Value	*FDR q*-Value
*Amino Acid Metabolism*			
	Valine	<0.001	<0.001
	Norvaline	<0.001	<0.001
	Histidine	<0.001	0.001
	Leucine	<0.001	0.004
	Isoleucine	<0.001	0.002
	Tyrosine	0.002	0.015
	Adenosyl-L-homocysteine	0.008	0.048
	Kynurenine	<0.001	0.005
	5-Hydroxyindoleacetic acid	0.004	0.031
	2-Aminoadipic acid	0.002	0.013
*Sugar Metabolism*			
	Pyruvate	<0.001	<0.001
	Glucose	<0.001	0.003
	Fructose	<0.001	0.003
	Mannose	<0.001	0.003
	Oxoglutaric acid	0.002	0.013
*Lipid Metabolism*			
	Propionyl-L-carnitine	<0.001	0.003
	2-Methylbutyryl-L-carnitine	0.008	0.048
	Isovaleryl-L-carnitine	0.007	0.046
	Isobutyric acid	<0.001	0.001
	2-Methylglutaric acid	<0.001	0.003
*Other Metabolism*			
	TMAO	<0.001	0.007
	Epinephrine	0.005	0.036
	Acetohydroxamic acid	<0.001	0.003
	N,N-Dicyclohexylurea	<0.001	0.001

Note. Data was calculated using a general linear model controlled for time and the four variables had statistically significant difference between the Best Responder and Matched Control groups (Table 2). A False Discovery Rate (FDR) was applied, and *p*-values were considered significant at <0.05.

## Data Availability

The original contributions presented in this study are included in this article; further inquiries can be directed to the corresponding author.

## Supplementary Materials

The following supporting information can be downloaded at: https://www.mdpi.com/article/10.3390/metabo15020075/s1. Table S1: Study flow and discussion topics of parent study; Table S2: Metabolites for which 12-week change was significantly different between Best Responder and Matched Control groups; Figure S1: Heat map of metabolites for which 12-week change was significantly different between Best Responder and Matched Control groups; Figure S2: Cross-validation test of partial least squares-discriminant analysis (PLS-DA) model.
