# Supplementary material for: Metabolome Alterations Associated with Three-Month Sitting-Time Reduction Among Sedentary Postmenopausal Latinas with Cardiometabolic Disease Risk
Source: Metabolites. 2025 Jan 26;15(2):75. doi: 10.3390/metabo15020075 (PMC11857752; doi:10.3390/metabo15020075)
Supplement: Supplementary file 1 [file metabolites-15-00075-s001.zip › metabolites-3445517-supplementary.pdf]

Metabolome Alterations Associated with Three-Month Sitting Time Reduction Among Sedentary Postmenopausal Latinas with Cardiometabolic Disease Risk

Jeffrey S. Patterson[1], Paniz Jasbi[2], Yan Jin[1], Haiwei Gu[1], Matthew A. Allison[3], Chase Reuter[4], Brinda K. Rana[5], Loki Natarajan[5,6], Dorothy D. Sears*[2,3,5,6]

[1]College of Health Solutions, Arizona State University, Phoenix, AZ, USA
[2]School of Molecular Science, Arizona State University, Phoenix, AZ, USA
[3]Department of Family Medicine, UC San Diego, La Jolla, CA, USA
[4]Department of Psychiatry, UC San Diego, La Jolla, CA, USA
[5]Herbert Wertheim School of Public Health and Human Longevity Science, UC San Diego, La Jolla, CA, USA
[6]Moores Cancer Center, UC San Diego, La Jolla, CA, USA

Contents:

Table S1: Study Flow and Discussion Topics of Parent Study

Table S2: Metabolites for Which 12-week Change was Significantly Different Between Best Responder and Matched Control Groups

Figure S1: Heat Map of Metabolites for Which 12-week Change was Significantly Different Between Best Responder and Matched Control Groups

Figure S2: Cross Validation Test of Partial Least Squares-Discriminant Analysis (PLS-DA) Model.

Table S1: Study Flow and Discussion Topics of Parent Study.

Week	Description of Study Flow and Discussion Topics	
0	Screening Visit 1: In Person Consent and initial screening	
0	Screening Visit 2: Fasting Blood Draw and activPAL collection	
	**Standing Intervention**	**Heart Healthy Lifestyle Comparison Control**
1	Add 30 min of standing in 5 min bouts/day from baseline	Caregiver and Stress
2	Add 60 min of standing in 5 min bouts/day from baseline	Insomnia and Sleep
3	Add 90 min of standing in 5 min bouts/day from baseline	Bladder Control
4	Add 120 min of standing in 5-10 min bouts/day from baseline	Home Safety
5	activPAL Progress Check	
6	Maintaining Goal of 120 min/day	Hydration
8	Maintaining Goal of 120 min/day	Depression & Aging
10	Maintaining Goal of 120 min/day	Stress Management
12	Maintaining Long-term Goals	Medication Management
13	Fasting Blood Draw and activPAL collection	

Note. Overview of 12-week study flow and discussion topics for groups in parent study.[44,45]

Table S2: Metabolites for Which 12-week Change was Significantly Different Between Best Responder and Matched Control Groups.

Metabolite	*P*-Value	*P*-Value (FDR)
Valine	1.53E-06	0.00021432
Pyruvate	8.90E-06	0.00062324
Norvaline	1.76E-05	0.00082144
Isobutyric acid	3.36E-05	0.0010431
N,N-Dicyclohexylurea	3.73E-05	0.0010431
Histidine	5.46E-05	0.0012739
Isoleucine	0.00012169	0.0024339
Glucose	0.0001884	0.0029675
Fructose	0.00019369	0.0029675
Propionyl-L-carnitine	0.00022595	0.0029675
Acetohydroxamic acid	0.00023892	0.0029675
Mannose	0.00027271	0.0029675
2-Methylglutaric acid	0.00027556	0.0029675
Leucine	0.00036441	0.0036441
Kynurenine	0.00053671	0.0050093
TMAO	0.00079254	0.0069348
2-Aminoadipic acid	0.0016816	0.013103
Oxoglutaric acid	0.0016846	0.013103
Tyrosine	0.002081	0.015333
5-Hydroxyindoleacetic acid	0.0043725	0.030608
Epinephrine	0.0053531	0.035687
Isovaleryl-L-carnitine	0.0071539	0.045525
2-Methylbutyryl-L-carnitine	0.0078413	0.04773
Adenosyl-L-homocysteine	0.0082257	0.047983
Nicotinamide	0.010475	0.058661
Amiloride	0.01268	0.06828
Agmatine	0.01549	0.080318
Picolinic acid	0.018476	0.087891
2,3-Dihydroxybenzoic acid	0.018643	0.087891
Protocatechuic acid	0.018834	0.087891
Glyoxylic acid	0.027034	0.12209
N-Acetylneuraminic acid	0.031641	0.12914
Hexanoyl-L-carnitine	0.032111	0.12914
Pentadecanoic acid	0.033313	0.12914
Valeric acid	0.033567	0.12914
Isovaleric acid	0.033782	0.12914

O-Acetyl-L-carnitine	0.034877	0.12914
Glycocyamine	0.035053	0.12914
Asparagine	0.041333	0.14837
Sorbitol	0.045035	0.15762
Homocysteine	0.049975	0.16955

Note. Data was calculated using a general linear model controlled for time and the four variables statistically significant different between the Best Responder and Matched Control groups (Table 2). A False Discovery Rate (FDR) was applied and *P*-values were considered significant at <0.05.

Figure S1:

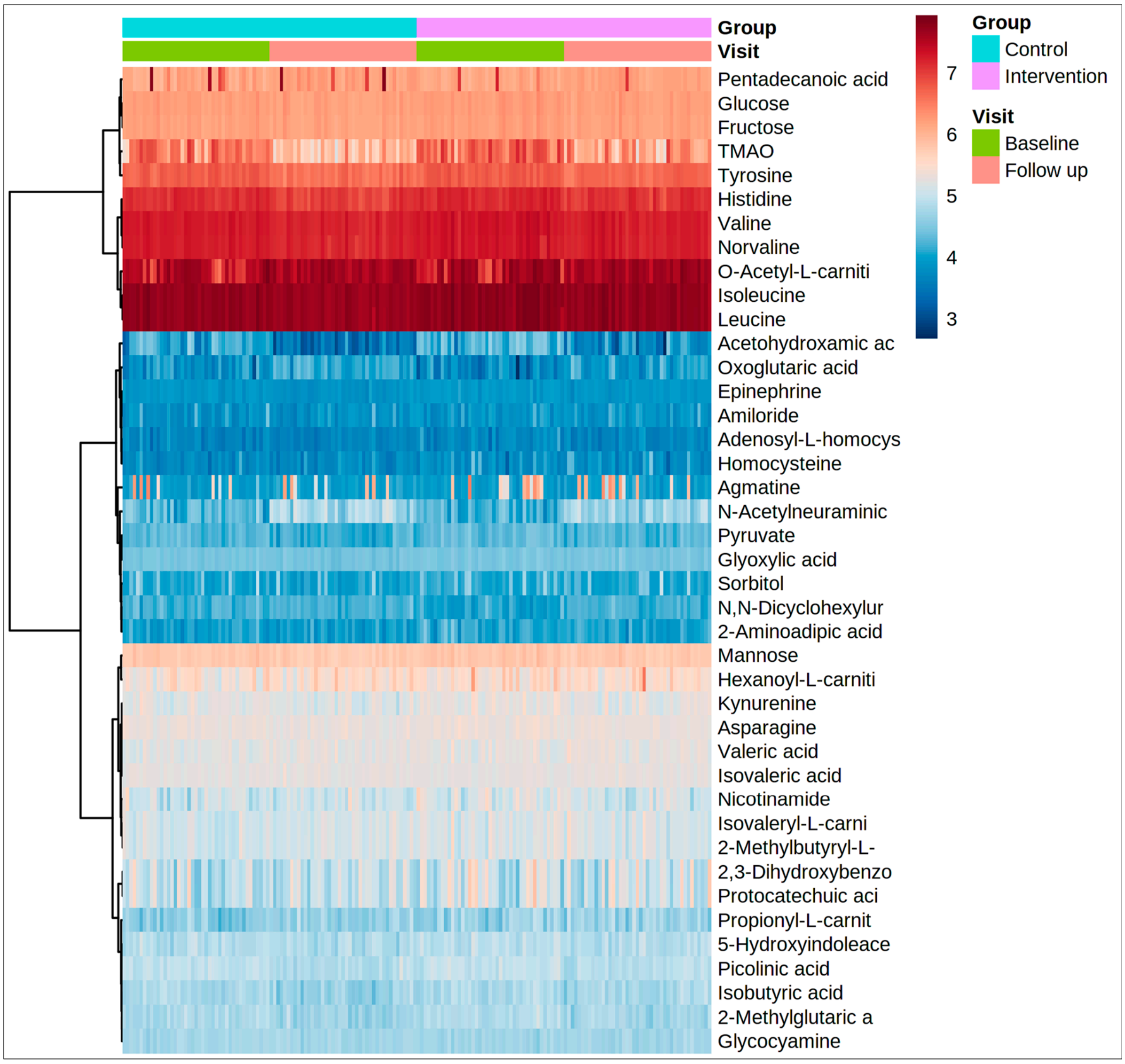

Figure S1. Heat Map of Metabolites for Which 12-week Change was Significantly Different Between Best Responder and Matched Control Groups. Display demonstrates normalized relative increases and decreases in abundance between and within conditions among postmenopausal women.

Figure S2:

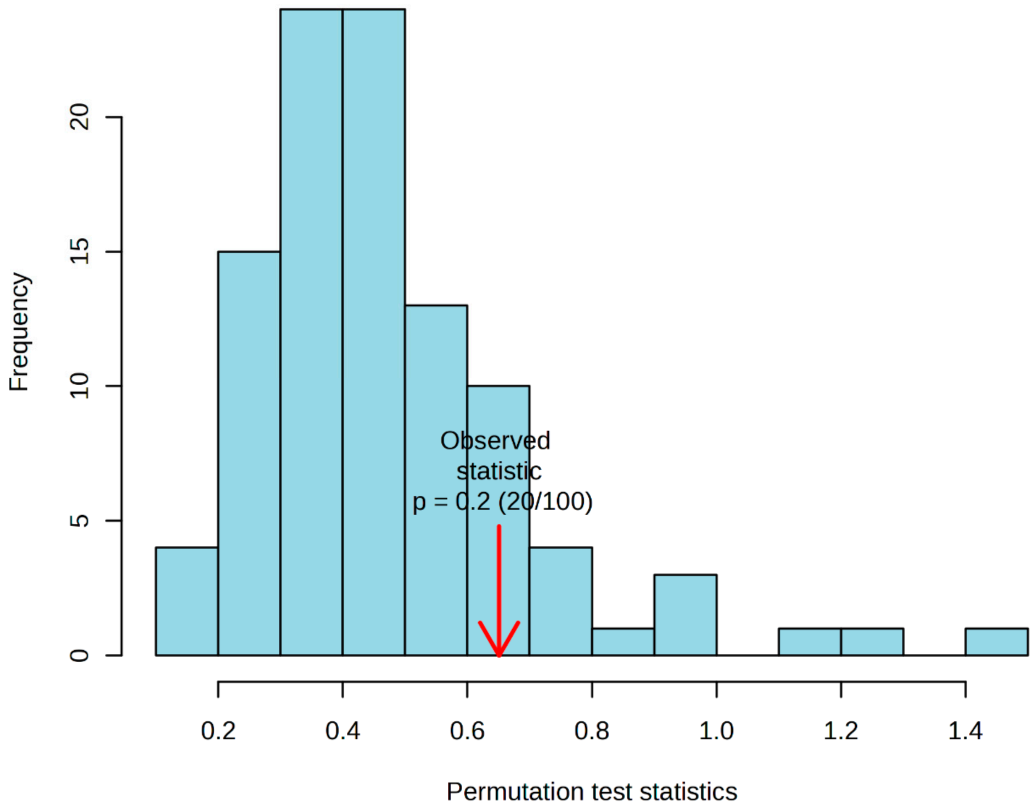

Figure S2. **Cross Validation Test of Partial Least Squares-Discriminant Analysis (PLS-DA) Model**. Cross validation of the PLS-DA was performed using permutation testing to ensure the model did not overfit the data. The observed statistic of the permutations was *P* = 0.20 (20/100).
